# Application of the Generalized Method of Moving Coordinates to Calculating Stress Fields near an Elliptical Hole

**DOI:** 10.3390/ma15186266

**Published:** 2022-09-09

**Authors:** Sergei Alexandrov, Marina Rynkovskaya, Shang-Nan Tsai

**Affiliations:** 1 Ishlinsky Institute for Problems in Mechanics RAS, 101-1 Prospect Vernadskogo, 119526 Moscow, Russia; 2 Department of Civil Engineering, Peoples’ Friendship University of Russia (RUDN University), 6 Miklukho-Maklaya St, 117198 Moscow, Russia; 3 Department of Mechanical and Electro-Mechanical Engineering, National Sun Yat-sen University, No. 70 Lien-Hai Rd., Kaohsiung 804, Taiwan

**Keywords:** holes, stress fields, moving coordinates, plasticity

## Abstract

The distribution of stresses near holes is of great importance in fracture mechanics and material modeling. The present paper provides a general stress solution near a traction-free surface for an arbitrary piecewise linear yield criterion, assuming plane-strain conditions. The generalized method of moving coordinates is proven efficient in this case. In particular, the solution reduces to evaluating one ordinary integral. The boundary value problem solved is a Cauchy problem for a hyperbolic system of equations. Therefore, the stress solution in the plastic region is independent of other boundary conditions, though the occurrence of plastic yielding at a specific point is path-dependent. The general solution applies to calculating the stress field near an elliptic hole. It is shown that the parameter that controls the pressure-dependency of the yield criterion affects the stress field significantly. The aspect ratio is less significant as compared to that parameter. However, for a given material, the aspect ratio should also be considered to predict the stress field accurately, especially in the near vicinity of the hole. The solution reduces to an available solution for the pressure-independent yield criterion, which is a particular yield criterion of the considered class of yield criteria.

## 1. Introduction

The distribution of stresses near holes and cracks is of great importance in fracture mechanics and material modeling. A vast amount of literature is devoted to developing methods for finding such distributions using the theories of elasticity and plasticity. The linearly elastic stress field at the base of a sharp stationary crack has been found in [1]. This analysis has been extended to the plastic range in [2,3,4].

The importance of stress solutions near holes is twofold. Firstly, such solutions are required to understand the fracture process. Paper [5] has presented a stress solution in the plastic region around an elliptic hole. This solution can be used in conjunction with the ductile fracture model proposed in [6]. Exact solutions for an elliptic hole embedded in a thermoelectric material have been presented in [7,8]. Paper [9] has found the stress distribution around an elliptic hole for non-local elastic material models. An elastic solution for a plate subject to biaxial loading and containing an inclined elliptic defect has been provided in [10]. The stress concentration generated by an elliptic hole in a functionally graded panel subjected to uniform tension has been analyzed in [11]. The effect of elastic anisotropy on the stress distribution around an elliptical crack has been revealed in [12], assuming that the medium is subjected to general loading at infinity. The influence of multiple pre-existing holes on the fracture process in granite subject to compressive loading has been studied in [13]. The stress concentration due to a circular hole in a plate subject to the uniaxial extension has been investigated in [14], using new constitutive equations with density-dependent elasticity moduli. This result has been extended to bi-axial deformation in [15]. A perturbation technique has been adopted in [16] for deriving analytical solutions for an elastic/plastic plate with a hole, including an elliptic hole. In particular, the elastic/plastic boundary has been found. Another analytical method for determining the elastic/plastic boundary in the vicinity of holes has been proposed in [17].

Another area of applications of the stress solutions near elliptic holes is that structural members contain such holes. Examples of such members have been considered in [18,19].

The present paper extends the solution [5] to an arbitrary piecewise linear yield criterion. Such yield criteria are widely used for describing metallic and nonmetallic materials [20,21,22]. The generalized method of moving coordinates is developed and used. The solution is semi-analytical. One needs to evaluate one ordinary integral to calculate the principal stresses at any point of the plastic region.

## 2. Basic Equations

Considered is plane-strain deformation of a material obeying an arbitrary piecewise linear yield criterion. Such yield criteria are represented by a linear function of the two principal stresses in a typical plane of flow. These stresses are denoted as $\sigma_{1}$ and $\sigma_{2}$. The yield criterion can be represented as:

(1) λp+q=1.

Here, λ is a constitutive parameter and

(2) $$p=-\frac{\sigma_{1}+\sigma_{2}}{2}\;\mathrm{and}\;q=\frac{\sigma_{1}-\sigma_{2}}{2}.$$

Note that Equation (1) represents the totality of piecewise linear yield criteria if the stresses are non-dimensionalized using an appropriate reference stress. For example, a standard form of the Mohr–Coulomb yield criterion is [23]:

(3) −psinχ+q=kcosχ

where the cohesion *k* and angle of internal friction χ are constants. Equation (3) reduces to Equation (1) if λ=−sinχ and the reference stress is kcosχ. It is also possible to choose without loss of generality that $\sigma_{1}>\sigma_{2}$. Then, q>0.

Let ψ be the inclination of the direction of the stress $\sigma_{1}$ relative to the $x_{1}-$axis of a Cartesian coordinate system $\left(x_{1},\;y_{1}\right)$, measured anticlockwise. Then, the stress components referred to the Cartesian coordinates are:

(4) $$\begin{array}{l} \sigma_{xx}=\frac{\left(\sigma_{1}+\sigma_{2}\right)}{2}+\frac{\left(\sigma_{1}-\sigma_{2}\right)}{2}\cos2\psi ,\;\sigma_{yy}=\frac{\left(\sigma_{1}+\sigma_{2}\right)}{2}-\frac{\left(\sigma_{1}-\sigma_{2}\right)}{2}\cos2\psi ,\;\\ \sigma_{xy}=\frac{\left(\sigma_{1}-\sigma_{2}\right)}{2}\sin2\psi . \end{array}$$

Equations (1), (2) and (4) combine to give:

(5) $$\begin{array}{l} \sigma_{xx}=-p\left(1+\lambda \cos2\psi \right)+\cos2\psi ,\;\\ \sigma_{yy}=-p\left(1+\lambda \cos2\psi \right)-\cos2\psi ,\;\\ \sigma_{xy}=\left(1-\lambda p\right)\sin2\psi . \end{array}$$

Equation (1) and the equilibrium equations comprise a statically determinate system. This system, without the assumption that the yield criterion is piecewise linear, has been analyzed in [24]. In the case of Equation (1), this analysis provides the following characteristics:

(6) $$\frac{dy_{1}}{dx_{1}}=\tan\left(\psi -\phi \right)\;\mathrm{and}\;\frac{dy_{1}}{dx_{1}}=\tan\left(\psi +\phi \right),$$

which are termed the α− and β−lines, respectively, where

(7) $$\tan\phi =\sqrt{\frac{1-\lambda }{1+\lambda }}.$$

The relations along the characteristics are:

(8) $$\sqrt{1-\lambda^{2}}dp+2\left(1-\lambda p\right)d\psi =0\;\mathrm{and}\;\sqrt{1-\lambda^{2}}dp-2\left(1-\lambda p\right)d\psi =0$$

on the α− and β−lines, respectively. It is seen from Equations (6) and (7) that the angle between the direction of the stress $\sigma_{1}$ and each characteristic line is constant. Therefore, Equation (8) can be rewritten as:

(9) $$\sqrt{1-\lambda^{2}}dp+2\left(1-\lambda p\right)d\varphi =0\;\mathrm{and}\;\sqrt{1-\lambda^{2}}dp-2\left(1-\lambda p\right)d\varphi =0$$

where

(10) φ=ψ−ϕ

is the angle between the x−axis and the α−characteristic line measured from the axis anticlockwise. If both families of characteristics are curved, the equations in (9) can be immediately integrated to give:

(11) $$P-\varphi =-2\alpha \cos\phi -\varphi_{0}\;\mathrm{and}\;P+\varphi =2\beta \cos\phi +\varphi_{0}$$

where

(12) $$P=-\frac{\sqrt{1-\lambda^{2}}}{2\lambda }\ln\left(\frac{1-\lambda p}{1-\lambda p_{0}}\right).$$

The constants $\varphi_{0}$ and $p_{0}$ have been introduced for further convenience. Note that Equation (12) reduces to $P=\left(p-p_{0}\right)/2$ as λ→0. Solving the equations in (11) for P and φ yields:

(13) $$P=\left(\beta -\alpha \right)\sin2\phi \;\mathrm{and}\;\varphi =\left(\beta +\alpha \right)\sin2\phi +\varphi_{0}.$$

## 3. Generalized Moving Coordinates

**Figure 1:**
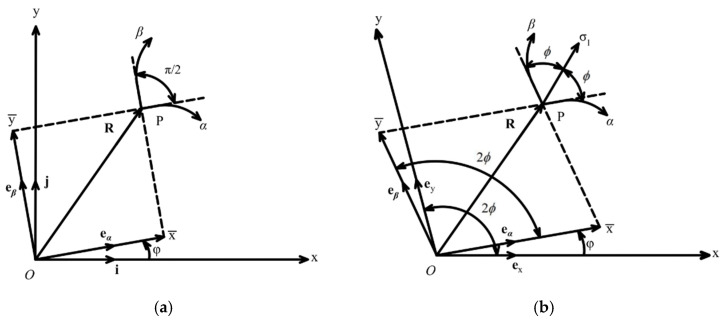
Moving coordinates: (**a**) pressure-independent yield criterion, (**b**) pressure-dependent yield criterion.

The moving coordinates $\left(\bar{x},\;\bar{y}\right)$ are used for solving boundary value problems in pressure-independent plasticity. This method has been described in many monographs, for example, [24]. In short, $\bar{x}$ and $\bar{y}$ are the coordinates of the point *C* under consideration, referred to axes passing through the origin *O* and parallel to the characteristic directions at *C*. The geometric interpretation of the moving coordinates is shown in Figure 1a. In the case of pressure-independent plasticity, the characteristic directions are orthogonal. Therefore, the moving coordinates are orthogonal. It is seen from Equations (6) and (7) that the characteristic directions are not orthogonal if λ≠0.

The geometric interpretation of the generalized moving coordinates, which are also denoted as $(\overset{-}{x},\overset{-}{y}),$ is shown in Figure 1b. In this figure, (x, y) is a skew rectilinear coordinate system with the origin at *O*. The x−axis is tangent to the base α−characteristic line passing through *O*, and the y−axis is tangent to the base β−characteristic line passing through *O*. Let $e_{x}$ and $e_{y}$ be the unit base vectors of the (x, y)−coordinate system. The unit vectors $e_{\alpha }$ and $e_{\beta }$ are directed along the α− and β−characteristic lines at the point *C* under consideration. Therefore, the angle between the vectors $e_{x}$ and $e_{\alpha }$ is φ. Then (Figure 1b):

(14) $$\begin{array}{l} e_{\alpha }\cdot e_{x}=\cos\varphi ,\;e_{\alpha }\cdot e_{y}=\cos\left(\varphi -2\phi \right),\;e_{\beta }\cdot e_{x}=\cos\left(\varphi +2\phi \right),\;\\ e_{\beta }\cdot e_{y}=\cos\varphi ,\;e_{y}\cdot e_{x}=\cos2\phi . \end{array}$$

The position vector of any point can be represented as

(15) $$R=xe_{x}+ye_{y}=\bar{x}e_{\alpha }+\bar{y}e_{\beta }.$$

Using Equations (14) and (15), one can find the scalar products, $R\cdot e_{x}$ and $R\cdot e_{y}$, as:

(16) $$\begin{array}{l} R\cdot e_{x}=x+y\cos2\phi =\bar{x}\cos\varphi +\bar{y}\cos\left(\varphi +2\phi \right),\\ R\cdot e_{y}=x\cos2\phi +y=\bar{x}\cos\left(\varphi -2\phi \right)+\bar{y}\cos\varphi . \end{array}$$

Solving these equations for $\bar{x}$ and $\bar{y}$ yields:

(17) $$\begin{array}{l} \bar{x}=x\frac{\left[\cos\varphi -\cos2\phi \cos\left(\varphi +2\phi \right)\right]}{\sin^{2}2\phi }+y\frac{\left[\cos\varphi \cos2\phi -\cos\left(\varphi +2\phi \right)\right]}{\sin^{2}2\phi },\\ \bar{y}=x\frac{\left[\cos\varphi \cos2\phi -\cos\left(\varphi -2\phi \right)\right]}{\sin^{2}2\phi }+y\frac{\left[\cos\varphi -\cos2\phi \cos\left(\varphi -2\phi \right)\right]}{\sin^{2}2\phi }. \end{array}$$

Alternatively, the equations in (16) can be solved for x and y. As a result,

(18) $$\begin{array}{l} x=\bar{x}\frac{\left[\cos\varphi -\cos2\phi \cos\left(\varphi -2\phi \right)\right]}{\sin^{2}2\phi }+\bar{y}\frac{\left[\cos\left(\varphi +2\phi \right)-\cos\varphi \cos2\phi \right]}{\sin^{2}2\phi },\\ y=\bar{x}\frac{\left[\cos\left(\varphi -2\phi \right)-\cos\varphi \cos2\phi \right]}{\sin^{2}2\phi }+\bar{y}\frac{\left[\cos\varphi -\cos2\phi \cos\left(\varphi +2\phi \right)\right]}{\sin^{2}2\phi }. \end{array}$$

Differentiating the first equation in (17) with respect to β and the second with respect to α leads to:

(19) $$\begin{array}{l} \sin^{2}2\phi \frac{\partial \bar{x}}{\partial \beta }=\left[\cos\varphi -\cos2\phi \cos\left(\varphi +2\phi \right)\right]\frac{\partial x}{\partial \beta }+\left[\cos\varphi \cos2\phi -\cos\left(\varphi +2\phi \right)\right]\frac{\partial y}{\partial \beta }-\\ \left\{x\left[\sin\varphi -\cos2\phi \sin\left(\varphi +2\phi \right)\right]+y\left[\sin\varphi \cos2\phi -\sin\left(\varphi +2\phi \right)\right]\right\}\frac{\partial \varphi }{\partial \beta },\\ \sin^{2}2\phi \frac{\partial \bar{y}}{\partial \alpha }=\left[\cos\varphi \cos2\phi -\cos\left(\varphi -2\phi \right)\right]\frac{\partial x}{\partial \alpha }+\left[\cos\varphi -\cos2\phi \cos\left(\varphi -2\phi \right)\right]\frac{\partial y}{\partial \alpha }-\\ \left\{x\left[\sin\varphi \cos2\phi -\sin\left(\varphi -2\phi \right)\right]+y\left[\sin\varphi -\cos2\phi \sin\left(\varphi -2\phi \right)\right]\right\}\frac{\partial \varphi }{\partial \alpha }. \end{array}$$

It is seen from Equation (13) that ∂φ/∂α=∂φ/∂β=sin2ϕ. Therefore, Equation (19) becomes:

(20) $$\begin{array}{l} \frac{\partial \bar{x}}{\partial \beta }=\frac{\left[\cos\varphi -\cos2\phi \cos\left(\varphi +2\phi \right)\right]}{\sin^{2}2\phi }\frac{\partial x}{\partial \beta }+\frac{\left[\cos\varphi \cos2\phi -\cos\left(\varphi +2\phi \right)\right]}{\sin^{2}2\phi }\frac{\partial y}{\partial \beta }-\\ \left\{x\left[\sin\varphi -\cos2\phi \sin\left(\varphi +2\phi \right)\right]+y\left[\sin\varphi \cos2\phi -\sin\left(\varphi +2\phi \right)\right]\right\}\csc2\phi ,\\ \frac{\partial \bar{y}}{\partial \alpha }=\frac{\left[\cos\varphi \cos2\phi -\cos\left(\varphi -2\phi \right)\right]}{\sin^{2}2\phi }\frac{\partial x}{\partial \alpha }+\frac{\left[\cos\varphi -\cos2\phi \cos\left(\varphi -2\phi \right)\right]}{\sin^{2}2\phi }\frac{\partial y}{\partial \alpha }-\\ \left\{x\left[\sin\varphi \cos2\phi -\sin\left(\varphi -2\phi \right)\right]+y\left[\sin\varphi -\cos2\phi \sin\left(\varphi -2\phi \right)\right]\right\}\csc2\phi . \end{array}$$

Using Equation (18), one can get after some trigonometry:

(21) $$\begin{array}{l} \left\{x\left[\sin\varphi -\cos2\phi \sin\left(\varphi +2\phi \right)\right]+y\left[\sin\varphi \cos2\phi -\sin\left(\varphi +2\phi \right)\right]\right\}\csc2\phi =-\left(\bar{y}+\bar{x}\cos2\phi \right),\\ \left\{x\left[\sin\varphi \cos2\phi -\sin\left(\varphi -2\phi \right)\right]+y\left[\sin\varphi -\cos2\phi \sin\left(\varphi -2\phi \right)\right]\right\}\csc2\phi =\bar{y}\cos2\phi +\bar{x}. \end{array}$$

Equation (6) can be rewritten using Equation (10) as:

(22) $$\frac{\partial y_{1}}{\partial \alpha }=\tan\varphi \frac{\partial x_{1}}{\partial \alpha }\;\mathrm{and}\;\frac{\partial y_{1}}{\partial \beta }=\tan\left(\varphi +2\phi \right)\frac{\partial x_{1}}{\partial \beta }.$$

**Figure 2:**
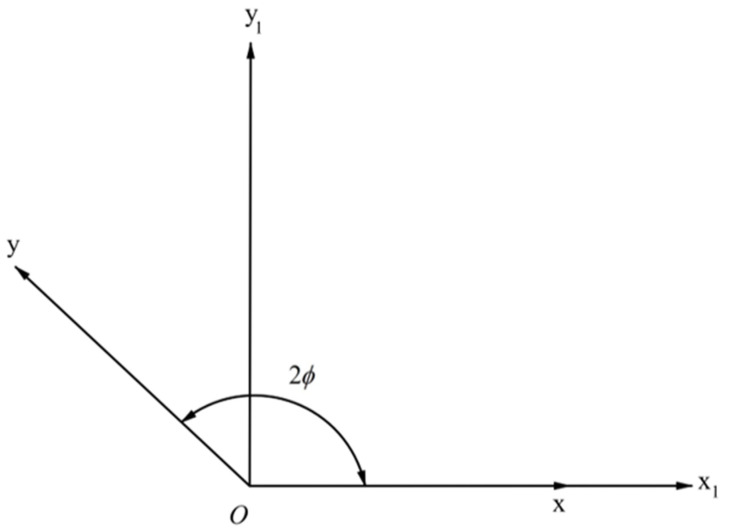
$\left(x_{1},\;y_{1}\right)-$coordinate system chosen for deriving Equation (28).

One can situate the origin of the $\left(x_{1},y_{1}\right)-$coordinate system at the origin of the (x,y)−coordinate system and direct the $x_{1}-$axis along the x−axis (Figure 2). Then,

(23) $$x=x_{1}-y_{1}\cot2\phi \;\mathrm{and}\;y=\frac{y_{1}}{\sin2\phi }.$$

Differentiating, one arrives at:

(24) $$\begin{array}{l} \frac{\partial x}{\partial \alpha }=\frac{\partial x_{1}}{\partial \alpha }+\frac{\partial y_{1}}{\partial \alpha }\cot2\phi ,\;\frac{\partial x}{\partial \beta }=\frac{\partial x_{1}}{\partial \beta }+\frac{\partial y_{1}}{\partial \beta }\cot2\phi ,\;\\ \frac{\partial y}{\partial \alpha }=\frac{1}{\sin2\phi }\frac{\partial y_{1}}{\partial \alpha },\;\;\frac{\partial y}{\partial \beta }=\frac{1}{\sin2\phi }\frac{\partial y_{1}}{\partial \beta }. \end{array}$$

Consider the terms of the equations in (20) containing the derivatives ∂x/∂α, ∂y/∂α, ∂x/∂β, and ∂y/∂β. Using Equations (22) and (24), one can represent these terms as:

(25) $$\begin{array}{l} \frac{\left[\cos\varphi \cos2\phi -\cos\left(\varphi -2\phi \right)\right]}{\sin^{2}2\phi }\frac{\partial x}{\partial \alpha }+\frac{\left[\cos\varphi -\cos2\phi \cos\left(\varphi -2\phi \right)\right]}{\sin^{2}2\phi }\frac{\partial y}{\partial \alpha }=\\ \left\{\begin{array}{l} \left[\cos\varphi \cos2\phi -\cos\left(\varphi -2\phi \right)\right]\left(\cot\varphi -\cos2\phi \right)+\\ \left[\cos\varphi -\cos2\phi \cos\left(\varphi -2\phi \right)\right]\csc2\phi \end{array}\right\}\csc^{2}2\phi \frac{\partial y_{1}}{\partial \alpha },\\ \frac{\left[\cos\varphi -\cos2\phi \cos\left(\varphi +2\phi \right)\right]}{\sin^{2}2\phi }\frac{\partial x}{\partial \beta }+\frac{\left[\cos\varphi \cos2\phi -\cos\left(\varphi +2\phi \right)\right]}{\sin^{2}2\phi }\frac{\partial y}{\partial \beta }=\\ \left\{\begin{array}{l} \left[\cos\varphi -\cos2\phi \cos\left(\varphi +2\phi \right)\right]\left[\cot\left(\varphi +2\phi \right)-\cot2\phi \right]+\\ \left[\cos\varphi \cos2\phi -\cos\left(\varphi +2\phi \right)\right]\csc2\phi \end{array}\right\}\csc^{2}2\phi \frac{\partial y_{1}}{\partial \alpha } \end{array}$$

Using trigonometric identities, one can show that the coefficient of $\partial y_{1}/\partial \alpha$ vanishes in each of these equations. Therefore, Equations (20) and (22) combine to give:

(26) $$\frac{\partial \bar{x}}{\partial \beta }-\left(\bar{y}+\bar{x}\cos2\phi \right)=0\;\mathrm{and}\;\frac{\partial \bar{y}}{\partial \alpha }+\bar{y}\cos2\phi +\bar{x}=0.$$

Introduce new variables $\bar{X}$ and $\bar{Y}$ as

(27) $$\bar{x}=\bar{X}\exp\left[\left(\beta -\alpha \right)\cos2\phi \right]\;\mathrm{and}\;\bar{y}=\bar{Y}\exp\left[\left(\beta -\alpha \right)\cos2\phi \right].$$

Substituting Equation (27) into Equation (26) yields:

(28) $$\frac{\partial \bar{X}}{\partial \beta }-\bar{Y}=0\;\mathrm{and}\;\frac{\partial \bar{Y}}{\partial \alpha }+\bar{X}=0.$$

It is seen from this equation that the quantities $\bar{X}$ and $\bar{Y}$ separately satisfy the equation of telegraphy. The latter is integrated by the method of Riemann.

## 4. Solution near a Traction-Free Surface

One of the principal stresses vanishes on any traction-free surface. The other principal stress and *p* are determined from Equations (1) and (2). It is necessary to consider two cases. One of these cases demands:

(29) $$\sigma_{1}=0,\;\sigma_{2}=-\frac{2}{1+\lambda },\;\mathrm{and}\;p=\frac{1}{1+\lambda }$$

on a traction-free surface. The other case demands:

(30) $$\sigma_{2}=0,\;\sigma_{1}=\frac{2}{1-\lambda },\;\mathrm{and}\;p=-\frac{1}{1-\lambda }$$

on a traction-free surface.

**Figure 3:**
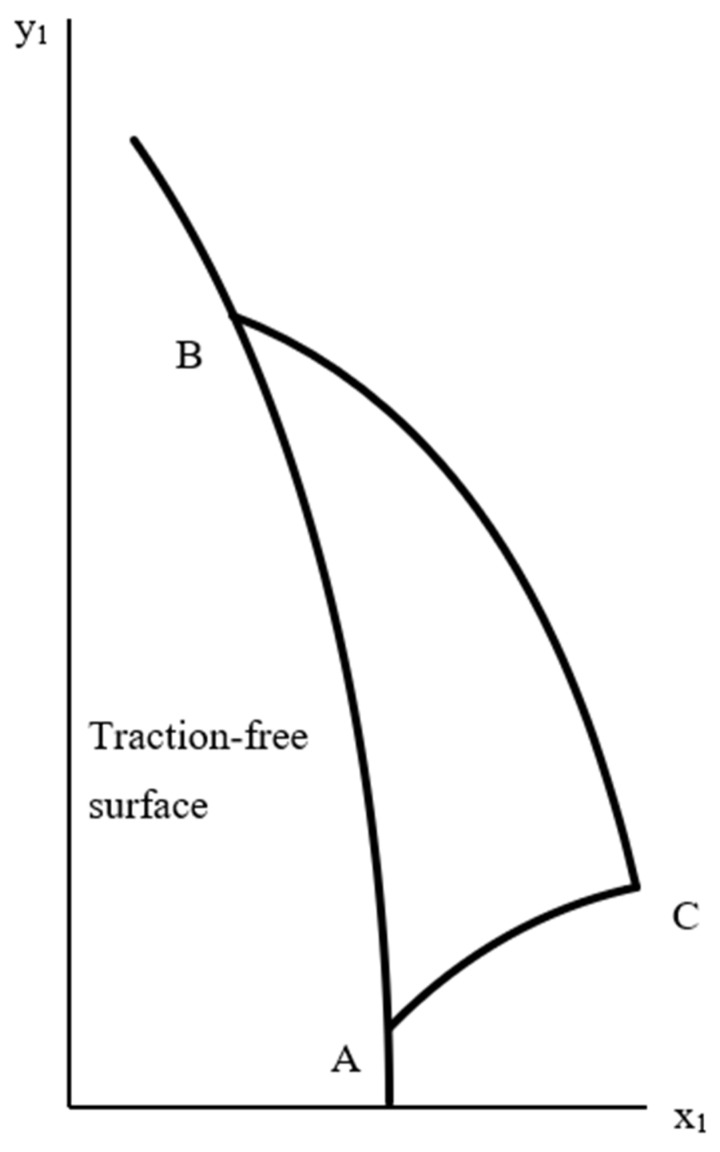
Illustration of the general boundary value problem.

Consider Equation (29). The general structure of the solution is shown in Figure 3. It is required to find $\bar{X}$ and $\bar{Y}$ at a point *C*. *BC* is an α−characteristic line, and *AC* is a β−characteristic line. Riemann’s method for the telegraph equation results in

(31) $$\oint_{BCA}\left(G\frac{\partial f}{\partial \alpha }-f\frac{\partial G}{\partial \alpha }\right)d\alpha +\left(f\frac{\partial G}{\partial \beta }-G\frac{\partial f}{\partial \beta }\right)d\beta =0.$$

Here *f* is $\bar{X}$ or $\bar{Y}$ and.

(32) $$G\left(\alpha_{C},\beta_{C},\alpha ,\beta \right)\equiv J_{0}\left[2\sqrt{\left(\alpha_{C}-\alpha \right)\left(\beta_{C}-\beta \right)}\right].$$

Also, $J_{0}\left(z\right)$ is the Bessel function of the first kind of zero order, $\alpha_{C}$ is the value of α at the point *C*, and $\beta_{C}$ is the value of β at the point *C*. Note that

(33) $$J_{0}\left(0\right)=1.\;\mathrm{and}\;J_{1}\left(0\right)=0$$

where $J_{1}\left(z\right)=-dJ_{0}\left(z\right)/dz$ is the Bessel function of the first kind of first order.

Since dβ=0 on *BC* and dα=0 on *AC*, Equation (31) can be rewritten as:

(34) $$\begin{array}{l} \oint_{BCA}\left(G\frac{\partial f}{\partial \alpha }-f\frac{\partial G}{\partial \alpha }\right)d\alpha +\left(f\frac{\partial G}{\partial \beta }-G\frac{\partial f}{\partial \beta }\right)d\beta =\int_{\alpha_{B}}^{\alpha_{C}}\left(G\frac{\partial f}{\partial \alpha }-f\frac{\partial G}{\partial \alpha }\right)d\alpha +\\ \int_{\beta_{C}}^{\beta_{A}}\left(f\frac{\partial G}{\partial \beta }-G\frac{\partial f}{\partial \beta }\right)d\beta +\int_{AB}\left(G\frac{\partial f}{\partial \alpha }-f\frac{\partial G}{\partial \alpha }\right)d\alpha +\left(f\frac{\partial G}{\partial \beta }-G\frac{\partial f}{\partial \beta }\right)d\beta =0. \end{array}$$

Moreover, $\beta =\beta_{C}$ on *BC* and $\alpha =\alpha_{C}$ on *AC*. Therefore, z=0 on these lines. Then, it follows from Equations (33) and (34) that:

(35) $$f_{C}=\frac{f_{B}+f_{A}}{2}-\frac{1}{2}\int_{AB}\left(G\frac{\partial f}{\partial \alpha }-f\frac{\partial G}{\partial \alpha }\right)d\alpha +\left(f\frac{\partial G}{\partial \beta }-G\frac{\partial f}{\partial \beta }\right)d\beta .$$

Here $f_{A}$, $f_{B}$, and $f_{C}$ are the value of *f* at *A*, *B*, and *C*, respectively.

Put $p_{0}=1/\left(1+\lambda \right)$ in (12). Then, it follows from Equations (12), (13), and (29) that:

(36) $$P=0,\;dP=0,\;d\beta =\frac{d\varphi }{2\sin2\phi },\;\mathrm{and}\;d\alpha =\frac{d\varphi }{2\sin2\phi }$$

on *AB*.

Using Equation (36), one can rewrite the integral in Equation (35) as

(37) $$\begin{array}{l} \int_{AB}\left(G\frac{\partial f}{\partial \alpha }-f\frac{\partial G}{\partial \alpha }\right)d\alpha +\left(f\frac{\partial G}{\partial \beta }-G\frac{\partial f}{\partial \beta }\right)d\beta =\\ =\frac{1}{2\sin2\phi }\int_{AB}\left(G\frac{\partial f}{\partial \alpha }-f\frac{\partial G}{\partial \alpha }+f\frac{\partial G}{\partial \beta }-G\frac{\partial f}{\partial \beta }\right)d\varphi . \end{array}$$

It follows from Equation (13) that:

(38) $$\frac{\partial }{\partial \alpha }=\sin2\phi \left(\frac{\partial }{\partial \varphi }-\frac{\partial }{\partial P}\right)\;\mathrm{and}\;\frac{\partial }{\partial \beta }=\sin2\phi \left(\frac{\partial }{\partial \varphi }+\frac{\partial }{\partial P}\right).$$

Equations (37) and (38) combine to give:

(39) $$\int_{AB}\left(G\frac{\partial f}{\partial \alpha }-f\frac{\partial G}{\partial \alpha }\right)d\alpha +\left(f\frac{\partial G}{\partial \beta }-G\frac{\partial f}{\partial \beta }\right)d\beta =\int_{AB}\left(f\frac{\partial G}{\partial P}-G\frac{\partial f}{\partial P}\right)d\varphi .$$

The derivative ∂f/∂P can be eliminated using Equation (28). In particular, it follows from Equations (28) and (38) that:

(40) $$\begin{array}{l} \frac{\partial \bar{Y}}{\partial P}=\frac{\partial \bar{Y}}{\partial \varphi }-\csc2\phi \frac{\partial \bar{Y}}{\partial \alpha }=\frac{\partial \bar{Y}}{\partial \varphi }+\bar{X}\csc2\phi ,\\ \frac{\partial \bar{X}}{\partial P}=\csc2\phi \frac{\partial \bar{X}}{\partial \beta }-\frac{\partial \bar{X}}{\partial \varphi }=\bar{Y}\csc2\phi -\frac{\partial \bar{X}}{\partial \varphi }. \end{array}$$

Moreover, by replacing α and β in Equation (32) with φ and *P* using Equation (13), one gets:

(41) $$G\left(\varphi_{C},P_{C},\varphi ,P\right)\equiv J_{0}\left(Z\right)$$

where $\varphi_{C}$ is the value of φ at *C*, $P_{C}$ is the value of *P* at *C*, and

(42) $$Z=\csc2\phi \sqrt{{\left(\varphi -\varphi_{C}\right)}^{2}-{\left(P-P_{C}\right)}^{2}}.$$

It follows from Equations (41) and (42) that:

(43) $$\frac{\partial G}{\partial P}=\frac{J_{1}\left(Z\right)\left(P-P_{C}\right)}{Z\sin^{2}2\phi }\;\mathrm{and}\;\frac{\partial G}{\partial \varphi }=-\frac{J_{1}\left(Z\right)\left(\varphi -\varphi_{C}\right)}{Z\sin^{2}2\phi }.$$

Substituting Equation (40) into Equation (39) and integrating by parts, one arrives at:

(44) $$\begin{array}{l} \int_{AB}\left(\bar{X}\frac{\partial G}{\partial P}-G\frac{\partial \bar{X}}{\partial P}\right)d\varphi =\int_{AB}\left[\bar{X}\left(\frac{\partial G}{\partial P}-\frac{\partial G}{\partial \varphi }\right)-G\bar{Y}\csc2\phi \right]d\varphi +{\bar{X}}_{B}-{\bar{X}}_{A},\\ \int_{AB}\left(\bar{Y}\frac{\partial G}{\partial P}-G\frac{\partial \bar{Y}}{\partial P}\right)d\varphi =\int_{AB}\left[\bar{Y}\left(\frac{\partial G}{\partial P}+\frac{\partial G}{\partial \varphi }\right)-G\bar{X}\csc2\phi \right]d\varphi -{\bar{Y}}_{B}+{\bar{Y}}_{A}. \end{array}$$

Eliminating here the derivatives ∂G/∂P and ∂G/∂φ using Equation (43) yields:

(45) $$\begin{array}{l} \int_{AB}\left(\bar{X}\frac{\partial G}{\partial P}-G\frac{\partial \bar{X}}{\partial P}\right)d\varphi =\csc2\phi \int_{AB}\left[\bar{X}\frac{J_{1}\left(Z\right)\left(\varphi -\varphi_{C}-P_{C}\right)}{Z\sin2\phi }-J_{0}\left(Z\right)\bar{Y}\right]d\varphi +{\bar{X}}_{B}-{\bar{X}}_{A},\\ \int_{AB}\left(\bar{Y}\frac{\partial G}{\partial P}-G\frac{\partial \bar{Y}}{\partial P}\right)d\varphi =-\csc2\phi \int_{AB}\left[\bar{Y}\frac{J_{1}\left(Z\right)\left(\varphi -\varphi_{C}+P_{C}\right)}{Z\sin2\phi }+J_{0}\left(Z\right)\bar{X}\right]d\varphi -{\bar{Y}}_{B}+{\bar{Y}}_{A}. \end{array}$$

Substituting Equation (45) into Equation (35) leads to:

(46) $$\begin{array}{l} {\bar{X}}_{C}={\bar{X}}_{A}-\frac{\csc2\phi }{2}\int_{\varphi_{A}}^{\varphi_{B}}\left[\bar{X}\frac{J_{1}\left(Z\right)\left(\varphi -\varphi_{C}-P_{C}\right)}{Z\sin2\phi }-J_{0}\left(Z\right)\bar{Y}\right]d\varphi ,\\ {\bar{Y}}_{C}={\bar{Y}}_{B}+\frac{\csc2\phi }{2}\int_{\varphi_{A}}^{\varphi_{B}}\left[\bar{Y}\frac{J_{1}\left(Z\right)\left(\varphi -\varphi_{C}+P_{C}\right)}{Z\sin2\phi }+J_{0}\left(Z\right)\bar{X}\right]d\varphi . \end{array}$$

The coefficients of $\bar{X}$ and $\bar{Y}$ in the integrands are known functions of φ due to Equations (36) and (42).

Consider Equation (30). In this case, *AC* is an α−characteristic line, and *BC* is a β−characteristic line (Figure 3). Putting $p_{0}=-1/\left(1+\lambda \right)$ in Equation (12) and repeating the line of reasoning that has led to Equation (46) in the previous case, one gets:

(47) $$\begin{array}{l} {\bar{X}}_{C}={\bar{X}}_{B}+\frac{\csc2\phi }{2}\int_{\varphi_{A}}^{\varphi_{B}}\left[\bar{X}\frac{J_{1}\left(Z\right)\left(\varphi -\varphi_{C}-P_{C}\right)}{Z\sin2\phi }-J_{0}\left(Z\right)\bar{Y}\right]d\varphi ,\\ {\bar{Y}}_{C}={\bar{Y}}_{A}-\frac{\csc2\phi }{2}\int_{\varphi_{A}}^{\varphi_{B}}\left[\bar{Y}\frac{J_{1}\left(Z\right)\left(\varphi -\varphi_{C}+P_{C}\right)}{Z\sin2\phi }+J_{0}\left(Z\right)\bar{X}\right]d\varphi . \end{array}$$

It follows from Equation (11) that:

(48) $$P_{C}=\frac{\varphi_{A}-\varphi_{B}}{2}\;\mathrm{and}\;\varphi_{C}=\frac{\varphi_{B}+\varphi_{A}}{2}$$

if Equation (29) is valid and

(49) $$P_{C}=\frac{\varphi_{B}-\varphi_{A}}{2}\;\mathrm{and}\;\varphi_{C}=\frac{\varphi_{B}+\varphi_{A}}{2}$$

Equation (30) is valid. Using Equations (48) and (49), one can eliminate $\varphi_{C}$ and $P_{C}$ in Equations (46) and (47).

The free surface geometry determines the quantities $\bar{X}$ and $\bar{Y}$ involved in Equations (46) and (47). Since P=0 on the free surface, it is seen from Equations (13) and (27) that:

(50) $$\bar{X}=\bar{x}\;\mathrm{and}\;\bar{Y}=\bar{y}$$

on *AB*. One of the principal stress directions is orthogonal to the free surface. Therefore, replacing φ with ψ in Equations (46) and (47) is convenient. It is seen from Equation (10) that dψ=dφ. Then, using Equations (48)–(50), one rewrites Equations (46) and (47) as:

(51) $$\begin{array}{l} {\bar{X}}_{C}={\bar{x}}_{A}-\frac{\csc2\phi }{2}\int_{\psi_{A}}^{\psi_{B}}\left[\bar{x}\frac{J_{1}\left(Z\right)\left(\psi -\psi_{B}\right)}{Z\sin2\phi }-J_{0}\left(Z\right)\bar{y}\right]d\psi ,\\ {\bar{Y}}_{C}={\bar{y}}_{B}+\frac{\csc2\phi }{2}\int_{\psi_{A}}^{\psi_{B}}\left[\bar{y}\frac{J_{1}\left(Z\right)\left(\psi -\psi_{A}\right)}{Z\sin2\phi }+J_{0}\left(Z\right)\bar{x}\right]d\psi , \end{array}$$

and

(52) $$\begin{array}{l} {\bar{X}}_{C}={\bar{x}}_{B}+\frac{\csc2\phi }{2}\int_{\psi_{A}}^{\psi_{B}}\left[\bar{x}\frac{J_{1}\left(Z\right)\left(\psi -\psi_{B}\right)}{Z\sin2\phi }-J_{0}\left(Z\right)\bar{y}\right]d\psi ,\\ {\bar{Y}}_{C}={\bar{y}}_{A}-\frac{\csc2\phi }{2}\int_{\psi_{A}}^{\psi_{B}}\left[\bar{y}\frac{J_{1}\left(Z\right)\left(\psi -\psi_{A}\right)}{Z\sin2\phi }+J_{0}\left(Z\right)\bar{x}\right]d\psi , \end{array}$$

respectively.

Equations (51) and (52) coincide with the solution [5] for pressure-independent material (i.e., ϕ=π/4).

## 5. Numerical Example

The distributions of $\bar{x}$ and $\bar{y}$ along *AB* are required to evaluate the integrals in Equations (51) and (52). The free surface shape determines these distributions. The numerical solution below is for an elliptic hole. Its equation can be written as:

(53) $$y_{1}^{2}+\frac{x_{1}^{2}}{a^{2}}=1\;\mathrm{or}\;\left(\sin^{2}\theta +\frac{\cos^{2}\theta }{a^{2}}\right)\rho^{2}=1$$

where $x_{1}=\rho \cos\theta$ and $y_{1}=\rho \sin\theta$. Equations (17) and (18) have been derived upon the assumption that the x−axis coincides with the $x_{1}-$axis. Therefore, the origin of the (x,y)−coordinate system should be situated at a point of the ellipse where the tangent to the α−is parallel to the $x_{1}-$axis. This condition is equivalent to:

(54) ψ=ϕ

at the origin of the (x,y)−coordinate system. Let $\theta_{0}$ be the value of θ at which this condition is satisfied. The components of the unit vector orthogonal to the ellipse are readily determined from Equation (53). As a result,

(55) $$a^{2}\tan\theta =\tan\psi .$$

Then, the condition Equation (54) yields:

(56) $$a^{2}\tan\theta_{0}=\tan\phi .$$

**Figure 4:**
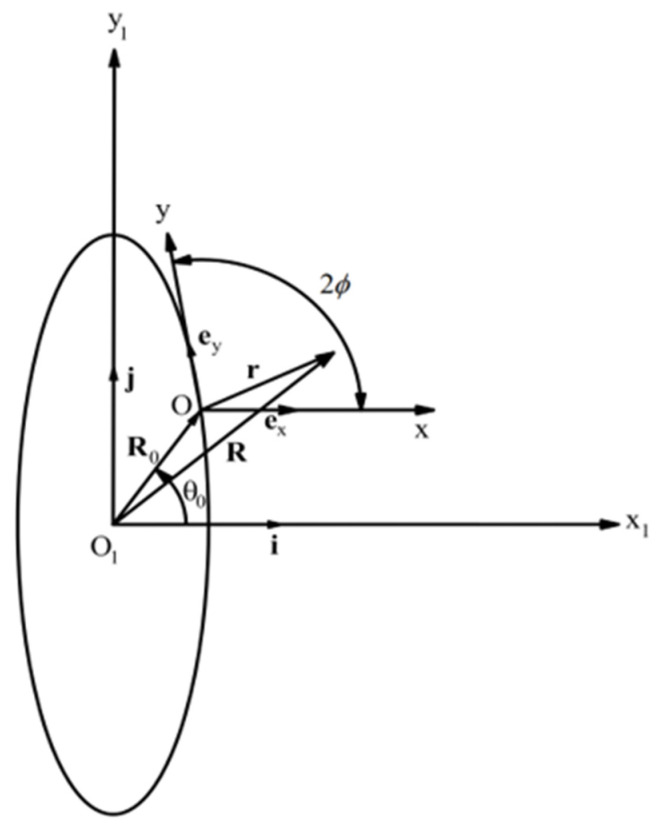
Illustration of the boundary value problem for an elliptic hole.

The ellipse, (x, y)−coordinate system, and $\left(x_{1},\;y_{1}\right)-$coordinate system are shown in Figure 4. The unit base vectors of the (x, y)−coordinate system are denoted as $e_{x}$ and $e_{y}$, and the unit base vectors of the $\left(x_{1},\;y_{1}\right)-$coordinate system as **i** and **j**. The position vector of a generic point is:

(57) $$R=R_{0}+r.$$

Using Equation (56), one can represent the vector $R_{0}$ as:

(58) $$\begin{array}{l} R_{0}=x_{0}i+y_{0}j,\\ x_{0}=\frac{a^{2}\cos\phi }{\sqrt{1-\left(1-a^{2}\right)\cos^{2}\phi }},\;y_{0}=\frac{\sin\phi }{\sqrt{1-\left(1-a^{2}\right)\cos^{2}\phi }}. \end{array}$$

It follows from the geometry of Figure 4 that:

(59) $$e_{x}\cdot i=1,\;e_{x}\cdot j=0,\;e_{y}\cdot i=\cos2\phi ,\;e_{y}\cdot j=\sin2\phi .$$

Using Equations (58) and (59), one can find the scalar products R⋅i and R⋅j from Equation (57) as:

(60) $$x_{1}=x_{0}+x+y\cos2\phi \;\mathrm{and}\;y_{1}=y_{0}+y\sin2\phi .$$

Solving these equations for *x* and *y* yields:

(61) $$x=x_{1}-y_{1}\cot2\phi -x_{0}+y_{0}\cot2\phi \;\mathrm{and}\;y=\frac{y_{1}-y_{0}}{\sin2\phi }.$$

It is convenient to rewrite these equations in terms of ρ and θ as

(62) $$x=\rho \left(\cos\theta -\sin\theta \cot2\phi \right)-x_{0}+y_{0}\cot2\phi \;\mathrm{and}\;y=\frac{\rho \sin\theta -y_{0}}{\sin2\phi }.$$

One can eliminate *r* in these equations using Equation (54). As a result,

(63) $$\begin{array}{l} x=\frac{a\left(\cos\theta -\sin\theta \cot2\phi \right)}{\sqrt{a^{2}\sin^{2}\theta +\cos^{2}\theta }}-x_{0}+y_{0}\cot2\phi ,\\ y=\frac{a\sin\theta }{\sin2\phi \sqrt{a^{2}\sin^{2}\theta +\cos^{2}\theta }}-\frac{y_{0}}{\sin2\phi }. \end{array}$$

**Figure 5:**
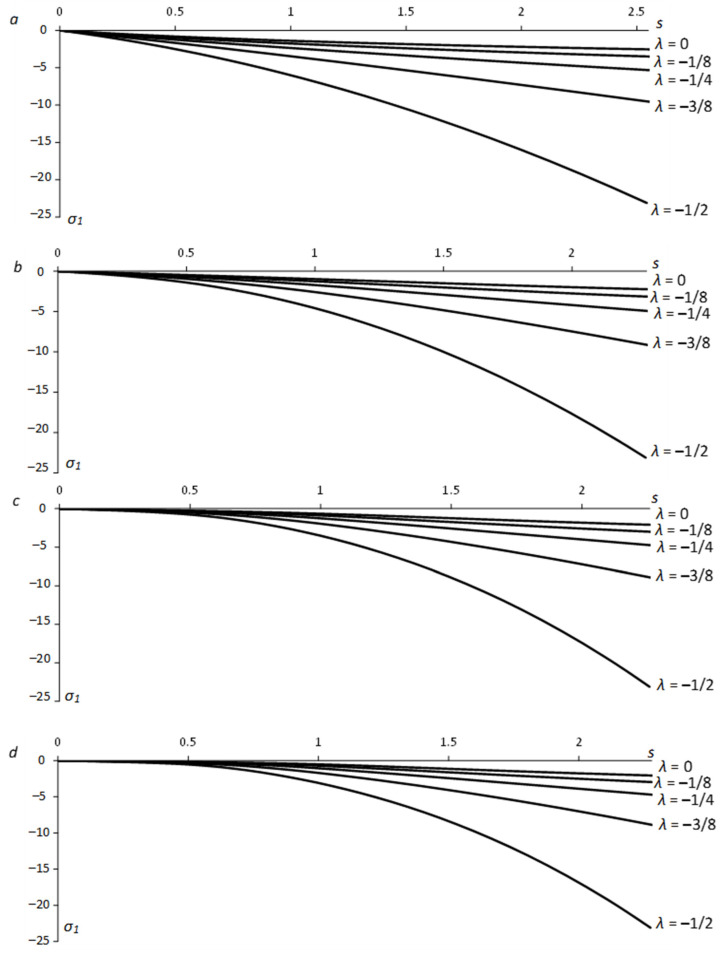
Dependence of the principal stress *σ*_1_ on the distance from the free surface along the *x*_1_-axis for several *λ*-values: (**a**) *a* = 1 (circular hole), (**b**) *a* = 1/2, (**c**) *a* = 1/4, (**d**) *a* = 1/6.

**Figure 6:**
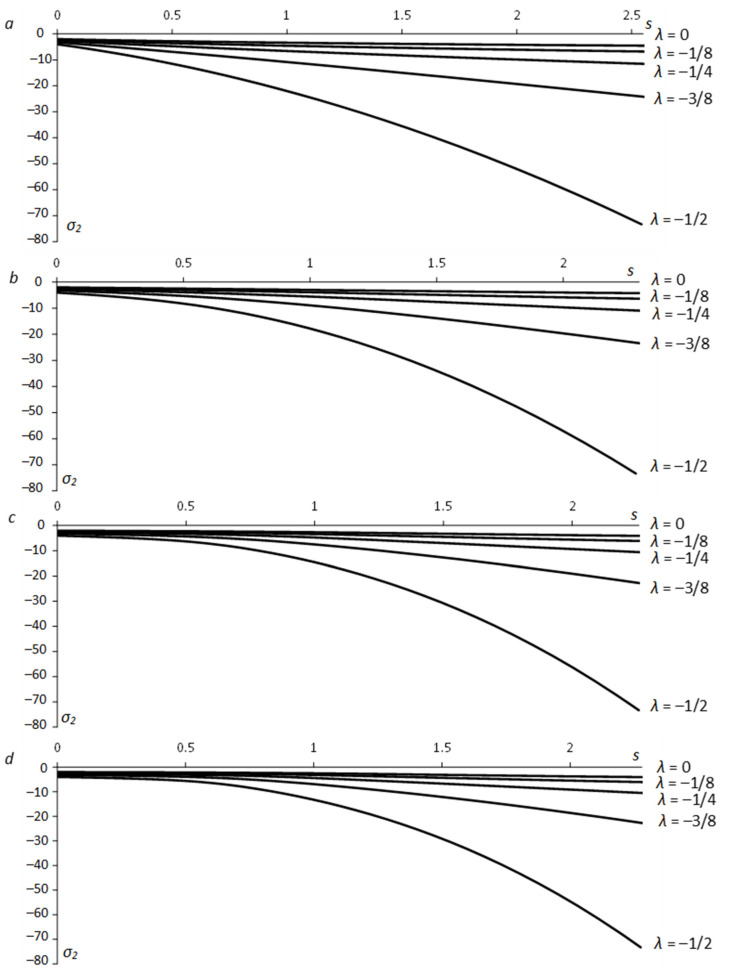
Dependence of the principal stress *σ*_2_ on the distance from the free surface along the *x*_1_-axis for several *λ*-values: (**a**) *a* = 1 (circular hole), (**b**) *a* = 1/2, (**c**) *a* = 1/4, (**d**) *a* = 1/6.

**Figure 7:**
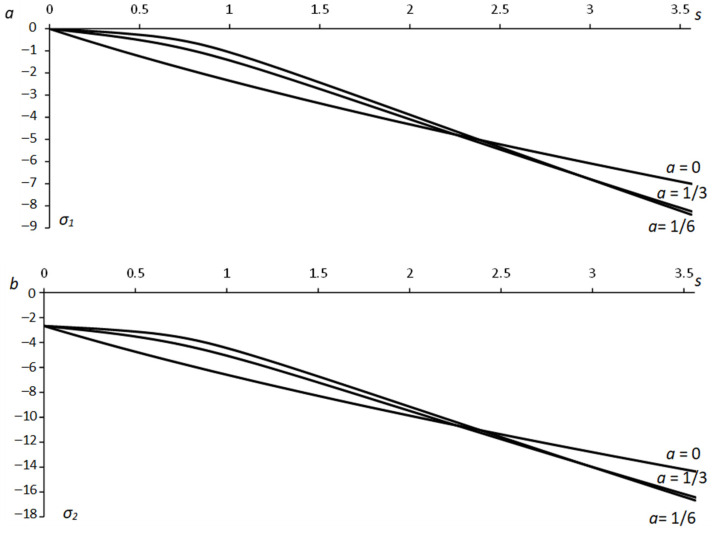
Dependence of the principal stress on the distance from the free surface along the *x*_1_-axis for several *a*-values at λ = −1/4: (**a**) *σ*_1_-stress, (**b**) *σ*_2_-stress.

Equation (55) allows for *x* and *y* to be calculated from Equation (63) as functions of ψ. Then, Equations (10) and (17) supply $\bar{x}$ and $\bar{y}$ as functions of ψ. These functions have been used in Equation (51) to calculate ${\bar{X}}_{C}$ and ${\bar{Y}}_{C}.$ Having ${\bar{X}}_{C}$ and ${\bar{Y}}_{C}$, one can calculate the $x_{1}-$ and $y_{1}-$coordinates of point *C* using Equations (18), (27), and (60). The principal stresses at this point are found from Equations (1), (2), (12), and (48) (or (49)). The numerical results below are for Equation (29). Figure 5 depicts the variation of the stress $\sigma_{1}$ along the $x_{1}-$axis for several values of λ and *a*. Figure 6 shows the variation of the stress $\sigma_{2}$. In these figures, *s* is the distance from the hole surface, $s=x_{1}-a$. Figure 5 and Figure 6 reveal a significant effect of λ on the stresses. In particular, the effect of *a* is invisible in these figures. However, the hole shape does affect the stresses near the surface. Figure 7 depicts the variation of the principal stresses for several *a*-values at λ=−1/4. It is seen from this figure that the value of $\left|\sigma_{1}\right|$ near a circular hole (a=1) is larger than its value near an elliptic hole. However, elliptical holes produce higher values of $\left|\sigma_{1}\right|$ than the corresponding circular hole at some distance from the hole’s surface. The same tendency occurs with the stress $\left|\sigma_{2}\right|$.

## 6. Conclusions

From this work, the following conclusions can be drawn:

(1) The generalized method of moving coordinates is efficient for determining stress fields near traction-free surfaces. In particular, determining the stress components at any point of the plastic region requires evaluating one ordinary integral in Equations (51) or (52).
(2) The solutions in Equations (51) and (52) are independent of other boundary conditions, though the shape of the plastic region is.
(3) The solution found is identical to the solution in [5] if *λ =* 0 in Equation (1).
(4) The parameter *λ* involved in Equation (1) has a much greater effect on the stress field than the aspect ratio (Figure 5 and Figure 6). However, it is a consequence of the yield criterion. For a given material, the aspect ratio affects the stress distribution in a small region near the hole (Figure 7).
(5) The solution found is for perfectly plastic material. However, slip-line solutions for the class of problems considered agree well with experimental results for strain-hardening materials [25].

## Data Availability

Not applicable.
